# Emerging roles of spexin in cardiovascular homeostasis

**DOI:** 10.3389/fcvm.2026.1704813

**Published:** 2026-02-18

**Authors:** Shuyun Wang, Jiangkang Xie, Zelin Li, Liang Mao, Xitong Dang

**Affiliations:** The Key Laboratory of Medical Electrophysiology, Ministry of Education, Institute of Cardiovascular Research, Department of Cardiology, The First Affiliated Hospital of Southwest Medical University, Southwest Medical University, Luzhou, Sichuan, China

**Keywords:** cardiovascular disease, diabetes, metabolic syndrome, neuropeptide, spexin

## Abstract

Spexin (SPX) is a highly conserved, proteolytically processed 14-amino acid peptide hormone derived from spexin precursor, originally identified through a bioinformatics-based prediction algorithm. SPX possesses pleiotropic effects across multiple organ system and has been implicated in the regulation of appetite, lipid and glucose metabolism, reproduction, inflammation, oxidative stress, neuropsychiatric functions, and cardiovascular physiology, with emerging evidence indicating sex-dependent effects in reproduction and metabolism. SPX exerts its biological effects via galanin receptors, engaging diverse G-protein coupled receptor signaling pathways. Altered SPX expression has been observed in a range of metabolic and systemic conditions, including obesity and metabolic syndrome, type 2 diabetes, hypertension, anxiety and depression, chronic kidney disease, non-alcoholic fatty liver disease, and aging, among others, highlighting its potential as both a diagnostic biomarker and a therapeutic target. Recent research suggests that SPX contributes to cardiovascular homeostasis by modulating autonomic regulation, inflammatory response, cardiomyocyte apoptosis, vascular tone, carotid body chemoreception, lipid metabolism, and insulin sensitivity, all of which are critical for maintaining cardiovascular health. This review concisely summarizes the distribution and regulation of SPX, with a focus on its emerging roles in the pathogenesis of cardiovascular disease and its potential as a theranostic agent.

## 1 Introduction

Cardiovascular disease (CVD) remains the leading cause of global morbidity and mortality, accounting for approximately 20 million deaths annually (1). According to the Global Burden of Disease 2019 study, this number is projected to reach 35.6 million by 2050 (2). It is well established that chronic inflammation, metabolic dysregulation including metabolic syndrome (MS), lipid abnormalities, obesity, along with aging, chronic stress, diabetes (DM), and neurohormonal imbalances, are key contributors to the development of CVD. Yet, existing therapies fall short in effectively targeting these fundamental mechanisms (3, 4) underscoring the urgent need for novel therapeutic targets that act earlier in the disease process. In this context, spexin (SPX), a highly conserved, 14-aminoacid (AA) peptide hormone derived from spexin precursor through proteolytically processing, has gained attention as a promising candidate due to its multifaceted regulatory functions, including modulation of metabolism, inflammation, cardiovascular homeostasis, endocrine signaling, stress adaptation, reproduction, and overall energy homeostasis (5–7).

Spexin precursor was first identified in 2007 by Mirabeau et al. through a bioinformatics-driven approach aimed at discovering novel peptide hormones encoded within the human genome. Using a hidden Markov model, they searched for short, conserved peptide sequences with features typical of secreted hormones, such as a signal peptide and conserved dibasic proteolytical cleavage sites. Two candidates were identified: Spexin and Augurin (8). Augurin, a 148-AA pre-pro-peptide encoded by chromosome 2 open reading frame 40 (C2ORF40), also known as esophageal cancer-related gene 4 (*ECRG4*), has been characterized not only as a tumor suppressor but also as a sentinel molecule involved in maintaining tissue homeostasis in various organs, including the heart (9–11). Spexin is a 116-AA pre-pro-peptide encoded by the gene initially annotated as C12ORF39, now recognized as the spexin gene (SPX) (12, 13).

Human *SPX*, approximately 220 kilobases (kb) in size, contains 6 exons and is mapped to chromosome 12p12.1. Transcription of *SPX* yields a 351-base pair (bp) ORF, encoding a 116-AA spexin precursor that includes a signal peptide (AA1–26) and three prohormone convertase sites (AA36–37, AA52–53, and AA72–73). Proteolytic processing of spexin precursor generates multiple small peptides, including a 14-AA mature form spexin (AA38–51, NWTPQAMLYLKGAQ, hereafter referred to as SPX) (14, 15). SPX, also known as neuropeptide Q (NPQ), shares a common evolutionary origin with galanin (GAL) and kisspeptin (KISS), despite their distinct physiological functions (16).

SPX is a highly conserved across species, playing a critical role in regulating fundamental physiological processes, including energy homeostasis, appetite, reproduction, stress, metabolism, and cardiovascular homeostasis (14, 17–22). SPX exerts its effects primarily through activation of GAL receptors GALR2 and GALR3, but not GALR1 (23). Dysregulation of SPX/GALR signaling has been implicated in metabolic disorders and the pathogenesis of various diseases (5, 24).

This review summarizes the distribution and regulation of spexin precursor, with a primary focus on the emerging roles of SPX in cardiometabolic homeostasis and its therapeutic potential in CVD associated with SPX-related chronic medical conditions, including obesity, diabetes (DM), metabolic syndrome (MS), and chronic renal failure (CRF).

## 2 Distribution and regulation of spexin

### 2.1 Spexin distribution and secretion

Spexin precursor is extensively expressed in both the central nervous system (CNS) and peripheral tissues. In the CNS, it has been detected in the hypothalamus, hippocampus, pituitary, and brainstem; while peripherally, it is expressed in the skin, lungs, gastrointestinal tract, liver, pancreas, endocrine system, kidneys, and cardiovascular system [carotid body (CB), heart, and vasculature], as well as in human term placenta (6, 8, 12, 25–28). Spexin precursor undergoes proteolytical processing within the canonical regulated secretory pathway, after which SPX is secreted into the circulation, supporting its classification as a peptide hormone with broad systemic effects (5, 29). Dysregulation of spexin precursor expression and/or SPX secretion has been implicated in the pathogenesis of various conditions, including anxiety and depression, diabetes, non-alcoholic fatty liver disease, MS, obesity, polycystic ovary syndrome (PCOS), CRF, and some CVDs (5).

### 2.2 Transcriptional regulation of spexin

Spexin precursor expression is regulated by nutritional, metabolic, hormonal, proinflammatory, psychological and environmental stress, and physical activity signals, reflecting its integrative role in maintaining energy balance and physiological homeostasis. Emerging evidence shows that these cues act at the transcriptional level. For example, Tran et al. demonstrated the presence of functional cis-elements for octamer-binding transcription factor-1 (OCT-1) and CCAAT/enhancer-binding protein beta (C/EBP-β) in the 5′ un-translated region of *Spx* gene. Notably, sodium nitroprusside [a well-known nitric oxide (NO) donor] enhanced the binding of C/EBP-β to these cis-elements (30). Mice subjected to chronic unpredictable stress triggered corticotropin-releasing factor (CRF)-induced trans-activation of the *Spx* gene via the adenylyl cyclase (AC)/cAMP and mitogen-activated protein kinase kinase 1 and 2 (MEK1/2)/extracellular signal-regulated kinase 1 and 2 (ERK1/2) signaling pathways, ultimately leading to suppressed *Spx* expression in the hippocampus (31). Additionally, intracerebroventricular (i.c.v.) injection of leptin decreased food intake and reduced body weight in mice. These effects were mediated by increased Spx in hypothalamic cells expressing the long isoform of the leptin receptor (ObRb, also known as obesity receptor, ObR) via signal transducer and activator of transcription 3 (STAT3)-dependent transactivation of the Spx gene (16). Together, these studies suggest that SPX gene expression is tightly regulated by diverse and context-dependent regulatory mechanisms.

### 2.3 Receptor-mediated mechanisms of SPX effect

Spexin exerts its diverse effects through binding to GALRs, engaging G protein–coupled receptors (GPCRs) (28). Binding to GALR2 is coupled to G protein *α*q/α11 subunit **(**Gq/11) subunits, leading to the activation of phospholipase C, an increase in intracellular calcium levels, and stimulation of protein kinase C (PKC). In contrast, binding to GALR3 is coupled to G protein *α* inhibitory/*α* other (olfactory) **(**Gi/o) subunits, which inhibits adenylate cyclase (AC) and subsequently reduces cyclic adenosine monophosphate (cAMP) levels (32, 33). Although downstream of signaling from GALRs remain poorly characterized, emerging evidence suggests context- and tissue-dependent mechanisms. For instance, palmitate, the most abundant saturated fatty acid in high-fat diet, upregulated the expression of *Spx*, *Galr2*, and *Galr3* mRNA in hypothalamic neuronal models involved in appetite regulation and reproduction. In appetite control, downstream signaling pathways primarily involved PKC and endoplasmic reticulum (ER) stress. By contrast, in reproductive regulation, pathways included toll-like receptor 4 (TLR4), PKC, c-Jun N-terminal kinase (JNK), extracellular signal-regulated kinase (ERK), and p38 mitogen-activated protein kinase (MAPK) (30, 34). In goldfish, postprandial increase in plasma spx and *spx* mRNA expression (liver and brain) were mediated via the insulin/insulin receptor signaling, activating both MAPK kinase 3/6-p38 MAPK and phosphoinositide 3-kinase (PI3K)/Akt (PKB)-mechanistic target of rapamycin (mTOR) cascades (20).

Functional studies further highlight receptor-dependent roles. Intraperitoneal Spx injection in mice significantly reduced cumulative food intake at 2-, 4-, and 6-h post-injection in fasted mice during the light period, and at 4 and 6 h in freely feeding mice during the dark period. These anorectic effects were mediated by hypothalamic GALR3 through downregulation of neuropeptide Y expression (6). In cardiomyocytes subjected to hypoxia, SPX reversed metabolic and mitochondrial dysfunction via GALR2, as these cardioprotective effects were abolished by M871(a GALR2 antagonist) (35). Similarly, in a rat model of high-fat/fructose diet (HFD)-induced metabolic dysfunction, Spx ameliorated weight gain, hyperglycemia, hyperinsulinemia, and insulin resistance (IR), confirming CALR2 involvement (36). Nevertheless, given SPX's pleiotropic actions across tissues and the potential existence of unidentified spexin-derived small peptides, the involvement of other receptors cannot be ruled out and warrants further investigation.

### 2.4 Regulation of SPX expression

#### 2.4.1 Metabolic and nutritional regulation

SPX expression responds to nutritional status in both fish and mammals. In Ya-fish, forebrain spx expression was significantly elevated post-prandially compared to pre-prandial levels, while fasting markedly suppressed its expression relative to fed controls (6, 37). Similarly, in goldfish, feeding increased plasma levels of glucose, insulin, and spx, along with elevated *spx* mRNA expression in the liver and appetite-regulatory regions of the brain. Intraperitoneal injections of glucose or insulin reproduced this effect, indicating positive regulation of spx by nutrient and insulin signaling. Mechanistically, insulin-mediated transcriptional activation of *spx* was confirmed in cultured hepatocytes and brain cells, which involved the insulin receptor/MAPK kinase 3/6/p38 MAPK and PI3K/Akt/mTOR signaling pathways (20).

In mammals, SPX is closely tied to metabolic status and has potential as both a diagnostic and prognostic biomarker for diabetes and other related metabolic disorders. Both *SPX* expression in adipose tissue and circulating SPX levels were reduced in adults with obesity and type 2 DM (T2DM), while decreased circulating SPX levels were also associated with obesity and IR in children and adolescents (38–41). One study exploring the dietary inflammatory index (DII) in obese children found that cardiometabolic alterations were driven primarily by changes in SPX levels, inflammation, and food intake rather than direct effects on metabolic syndrome components (42). Additionally, SPX was significantly down-regulated in first-degree relatives (FDR) of T2DM patients, as well as individuals with impaired glucose regulation (IGR) (43). In contrast, children with high fat mass and elevated systolic blood pressure (SBP) exhibited higher serum SPX levels than those with normal values, possibly reflecting a compensatory response. After adjustment for confounders, the highest tertile of serum SPX was independently associated with a protective effect against MS. Moreover, SPX levels were inversely correlated with key metabolic and cardiovascular risk factors, including total dietary fat intake, body mass index (BMI), triglycerides, total cholesterol, and fasting insulin, suggesting its role in lipid metabolism and glucose homeostasis (5, 44, 45). Notably, bariatric surgery restored SPX levels in obese patients to those of healthy controls, while exercise and a low-carbohydrate ketogenic diet also increased SPX expression (5, 46–48). Altogether, these findings establish SPX as a key regulator of metabolism and energy homeostasis, with potential as a promising target for the prevention and treatment of metabolic disorders.

#### 2.4.2 Hormonal regulation

Spexin precursor expression is modulated by hormonal signaling, aligning with its role in metabolic and reproductive regulation. While leptin and insulin downregulated its expression peripherally, evidence from the hypothalamus suggests a more nuanced interaction. In neurons of hypothalamus expressing the ObRb, leptin upregulated Spx via STAT3-dependent transcriptional activation. Inhibition of Spx biosynthesis in this setting attenuated leptin's anorexigenic effects and impaired downstream signaling to proopiomelanocortin (POMC) neurons, indicating that Spx acts as a key mediator of leptin's central regulatory actions (16). In the reproductive system, SPX acts as a negative regulator via the hypothalamic-pituitary-gonadal (HPG) axis. It inhibited the expression of gonadotropin-releasing hormone (GnRH) in the hypothalamus, luteinizing hormone (LH) and follicle-stimulating hormone (FSH) in the pituitary, and granulosa cell proliferation and estradiol (E2) release in the ovary (17, 49). Interestingly, E2, in turn, suppressed SPX expression in the hypothalamus in a dose-dependent manner (50), indicating a bidirectional negative feedback loop between SPX and sex hormones in female reproductive system. In contrast, SPX appears to have a stimulatory effect on the male HPG axis, with i.c.v. infusion increasing hypothalamic *GnRH* mRNA expression and elevating circulating LH, FSH, and testosterone levels independent of dosage. Histological analyses further reveal that SPX treatment increased seminiferous tubule diameter without altering epithelial thickness (18). These results indicate that SPX modulates the HPG axis in a sex-dependent manner.

#### 2.4.3 Stress-related regulation

SPX is regulated by social and psychological stress through stress-related hormones. In male teleosts subjected to chronic social defeat stress, spx1a (the evolutionarily conserved ortholog of human SPX) was upregulated. In primary brain cell cultures, serotonin (5-HT) downregulated spx1a expression, with 5-HT immunoreactive projections closely associated with spx1a neurons in the semicircular torus, where both immunoreactivity neurons and gene expression were significantly increased. Consistently, citalopram (a selective 5-HT reuptake inhibitor antidepressant) normalized *spx1a* expression to control levels, indicating that 5-HT acts as a negative upstream regulator of spx1a during social defeat (51). In mice subjected to chronic unpredictable stress, *Spx* mRNA levels in the hippocampus were significantly reduced, concomitant with corticotropin-releasing factor (CRF) mRNA upregulation. Direct hippocampal CRF injection suppressed *Spx* mRNA expression not only in the hippocampus but also in the hypothalamus and pituitary. This inhibitory action was replicated in primary hippocampal cell cultures and mediated via CRF receptor 2 (CRFR2)/AC/cAMP/PKA and MEK1/2/Erk1/2/Exchange Protein Directly Activated by cAMP (Epac) signaling pathways (31). These findings suggest that SPX participates in the neuroendocrine stress response and could function as a downstream effector in the hypothalamic-pituitary-adrenal (HPA) axis. In rats treated with the selective serotonin reuptake inhibitor (SSRI), escitalopram, for 4 weeks, *Spx* mRNA and Spx expression decreased in the hypothalamus but increased in the hippocampus and striatum, implying that SPX expression is modulated by SSRIs and may contribute to their neurobiological effects (52). While preclinical models strongly suggest an inverse relationship between Spx levels and social stress, human data remains inconclusive. In a cohort of 219 females, including 68 with anorexia nervosa, 79 with obesity, and 72 with normal weight, plasma SPX levels correlated negatively with BMI and body fat mass, but showed no association with perceived stress, anxiety, depressive symptoms, eating behavior, energy expenditure, or physical activity (53).

#### 2.4.4 The effect of exercise on SPX

A growing body of evidence highlights the positive impact of exercise on SPX levels and metabolic health in human and animal models. Both aerobic and resistance training consistently increased plasma SPX levels, with effects observed in elderly individuals, overweight/obese men, and patients with T2DM. However, data remain limited in healthy, normal-weight adults remain limited, underscoring the need for further research in this population. In rodent models, both acute and chronic exercise upregulated Spx levels, with the liver and skeletal muscle identified as potential sources of circulating Spx (46, 47, 54–56). These increases were often associated with improved insulin sensitivity, body composition, and lipid profiles, effects further enhanced by concomitant dietary interventions, such as vitamin D (VITD) or spirulina supplementation (47, 57). Exercise-induced SPX elevation appeared to modulate appetite, body fat, and inflammatory markers, mediated in part by GALR2/3 (54, 56). Furthermore, SPX promoted skeletal muscle cell proliferation and differentiation, supporting its role in metabolic regulation (56). Notably, individuals with higher BMI and adiposity exhibited lower baseline SPX levels, which were significantly elevated following structured exercise interventions, particularly in those with or at risk of developing T2DM (5, 47, 54, 55, 58–60). Nevertheless, variability exists on appetite post exercise. Acute exercise yielded in consistent appetite responses within 24 h, whereas chronic exercise generally maintained or a reduced appetite ratings compared to non-exercise controls, often accompanied by inconsistent SPX changes, suggesting context-dependent mechanisms in prediabetes or T2DM (60). Additionally, in a mixed cohort of normal-weight, obese, and anorexic females, plasma SPX levels negatively correlated with BMI and fat mass but showed no association with physical activity or energy expenditure, indicating involvement of other physiological or psychological factors (53). These inconsistencies highlight the context-dependent nature of SPX responses to exercise, influenced by factors such as sex, baseline metabolic status, exercise modality, and duration. Collectively, physical activity emerges as a potent modulator of SPX, contributing to broader metabolic improvements and appetite regulation, though individual variability warrants consideration in clinical and research settings.

#### 2.4.5 Pathological regulation and therapeutic implications

Epidemiological studies have shown that circulating SPX levels are negatively correlated with age, BMI, fasting blood glucose, IR, HbA1c, and triglycerides in healthy adult women. Consistently, SPX levels were reduced in obesity, T1DM and T2DM, and CVD, with even lower levels observed in diabetic patients with cardiovascular complications (44, 61–64). Experimental studies further indicate that exogenous SPX administration promoted weight loss, stimulated *β* cell proliferation and glucose-stimulated insulin secretion (GSIS), improved glycemic control, and suppressed hepatic glucose production (65, 66). In models of diabetic nephropathy, SPX treatment improved glucose metabolism, reduced serum urea and creatinine, lowered proinflammatory cytokines (IL-1β and TNF-α), and restored antioxidant enzyme activity. These beneficial effects were accompanied by downregulation of nuclear factor kappa-light-chain-enhancer of activated B cells (NF-κB), mTOR, and B-cell lymphoma 2 **(**Bcl-2)-associated X protein (Bax), while upregulating Bcl-2 and E-cadherin, aligning with improved renal histopathology (5, 66–69). These findings underscore SPX's therapeutic potential in metabolic dysfunction and its complications. A recent experimental study comparing the therapeutic effect of curcumin (CUR) and nano-CUR (nCUR) in a T2DM rat model demonstrated that both formulations significantly improved IR, fasting blood glucose, and lipid profiles. These improvements were accompanied by increased plasma Spx and hepatic *Spx* gene expression in, with nCUR producing a much more pronounced effect than CUR. These results suggest that Spx may mediate, at least in part, the therapeutic effect of curcumin in T2DM (70).

Interestingly, in gestational DM (GDM), circulating SPX levels were significantly elevated compared to healthy pregnant controls and positively correlated with IR, particularly during the third trimester (71). This dynamic expression pattern contrasts sharply with the downsregulation observed in non-GDM, suggesting that its expression and function may be context-dependent and influenced by the unique hormonal, metabolic, and inflammatory milieu of pregnancy. These observations underscore the need for disease-specific investigation of SPX signaling and raise the possibility of tailored therapeutic applications across different metabolic and cardiovascular disorders.

## 3 The roles of SPX in cardiovascular homeostasis

SPX regulates cardiovascular function through coordinated central and peripheral actions, positioning it as an integrative modulator of cardiovascular homeostasis.

Within the CNS, spexin precursor is expressed in several regions involved in autonomic and neuroendocrine regulation, including the hypothalamus (particularly the paraventricular and arcuate nuclei), brain stem structures such as Barrington's nucleus, locus coeruleus, laterodorsal tegmental nucleus, and caudal periaqueductal gray, as well as the choroid plexus. SPX frequently co-localizes with corticotropin-releasing hormone (CRH), tyrosine hydroxylase, or tryptophan hydroxylase (72), supporting the potential roles in mood, stress, arousal, and autonomic function. Additional sites of expression, such as retinal photoreceptors, Purkinje cells, and several hypothalamic nuclei, further underscore its broad neuroanatomical distribution (12, 14). Emerging evidence indicates that central SPX directly influences cardiovascular and renal function. Administration of Spx (i.c.v.) in rats increased mean arterial pressure (MAP), decreased HR, and reduced urine flow rate, suggesting activation of central autonomic and fluid balance pathways. In contrast, a distinct spexin peptide (AA53–70), elicited opposite effects, decreasing HR, leaving MAP unchanged, and markedly increased urine output (29). Taken together, these findings suggest that SPX, along with spexin (AA53-70), plays a crucial role in cardiovascular homeostasis by integrating central autonomic outflow to regulate HR, BP, and fluid balance.

Peripherally, SPX preserves cardiac function against insults such as ischemia and chemotherapeutic toxicity, influences renal handling of fluid and electrolytes (contributing to long-term BP control), modulates vascular tone and inflammation, and affects pulmonary and carotid body functions, thereby linking oxygen sensing and respiratory-cardiovascular coupling to autonomic regulation. Here, we summarize the primary peripheral actions of SPX in the pathogenesis of various CVDs (Table 1), while noting inconsistencies across studies.

**Table 1:** Summary of key functions of SPX on cardiovascular homeostasis.

Article	Key findings
Liu et al., 2020. Spexin protects cardiomyocytes from hypoxia-induced metabolic and mitochondrial dysfunction	Expressed in cardiac tissue and reduced by hypoxia; Enhances fatty acid metabolism; Preserves mitochondrial function and energy production during hypoxia.
Ou et al., 2024. Spexin inhibits excessive autophagy-induced ferroptosis to alleviate doxorubicin-induced cardiotoxicity	Protects against DOX-induced cardiotoxicity by suppressing excessive autophagy-dependent ferroptosis, likely via a Beclin-1-mediated mechanism.
Kumar et al., 2018. Spexin & cardiovascular risk markers in obese adolescents	Circulating SPX was inversely correlated with CRP levels
Said et al., 2023. Spexin alleviates hypertension, dyslipidemia, insulin resistance	Reduces BP, dyslipidemia, and inflammation via PPAR*γ* and AMPK activation
Ciftci et al., 2023. Spexin level in acute myocardial infarction	Lower SPX levels in STEMI and NSTEMI patients than controls, suggesting potential as a diagnostic marker in AMI.
Li et al., 2024. Spexin diminishes atrial fibrillation vulnerability via GALR2	Reduces AF susceptibility by modulating ion channel expression and Ca^2^^+^ handling via GALR2/CREB signaling.
Memi et al., 2025. Effects on cardiac inflammation & vascular response	Reduces CRF-induced vascular damage by decreasing CK-MB; Induces NO–dependent improvements in vascular reactivity; Downregulates inflammatory and extracellular matrix markers (ILs MMPs, and NGAL).
Kulualp et al., 2025. Exogenous spexin aggravates renal ischemia reperfusion injury and triggers toxicity in healthy kidneys	SPX treatment aggravates renal IRI by activating the Wnt/*β*-catenin signaling pathway; Promotes inflammation, apoptosis, and fibrosis; SPX exertes more severe deleterious nephrotoxic effects in healthy kidney than in IRI-injured kidney
Toll et al., 2012. Peptides derived from the prohormone proNPQ/spexin are potent central modulators of cardiovascular and renal function and nociception	SPX increases MAP; SPX decreases HR and urine flow; Spexin(53–70) decreases HR and increases urine flow
Onat et al., 2023. The protective effects of humanin in rats with experimental myocardial infarction: The role of asprosin and spexin	Decreases serum AST, LDH, CK-MB, and Troponin I; Reduces erythrocyte extravasation, congested veins, and necrotic muscle fibers.
Porzionato et al., 2012. Spexin is expressed in the carotid body and is upregulated by postnatal hyperoxia exposure	Expressed in Type I cells functioning as peripheral chemoreception; Hyperoxia induces SPX expression contributing to hyperoxia-induced structural plasticity of the CB
Tas et al., 2024. Comparison of Serum Spexin Level and its Relationship with Echocardiographic Findings in Prediabetic Patients with and without Hypertension	Low circulating SPX in hypertensive patients (especially non-dippers); Inverse correlation with nighttime BP; SPX attenuates hypertension in metabolic syndrome models

### 3.1 Myocardial ischemia/reperfusion injury

Spexin precursor is expressed in cardiomyocytes, endothelial cells, and epicardial adipose tissue (35). In humans, low circulating SPX levels were associated with increased CVD risk factors, including obesity, hypertension, dyslipidemia, and IR, particularly in adolescents and adults with MS (5, 73).

Accumulating evidence suggests that SPX plays a significant role in the pathophysiological response to myocardial ischemia/reperfusion injury (MIRI). In a rat model of isoproterenol-induced myocardial infarction (MI), cardiac Spx levels significantly increased by day 7 post-MI, consistent with a stress-associated response. However, pretreatment with humanin prior to MI resulted in significantly lower Spx levels compared to untreated controls. This reduction was accompanied by decreased infarct severity, vascular congestion, and myocardial necrosis (74). Mechanistically, SPX pretreatment H9C2 cells and neonatal rat cardiomyocytes (NRCMs) before hypoxia enhanced fatty acid metabolism and energy production via upregulation of key genes involved in fatty acid uptake and oxidation, including fatty acid translocase/cluster of differentiation 36 (FAT/CD36), carnitine palmitoyltransferase 1 (CPT1), Acyl-CoA dehydrogenase medium chain (ACADM), peroxisome proliferator-activated receptor alpha (PPAR-α), and peroxisome proliferator-activated receptor gamma coactivator 1*α* (PGC1*α*), while ameliorating hypoxia-induced downregulation of mitochondrial transcription factor A (TFAM) and electron transport chain (ETC) components, suppressing the expression of uncoupling protein 2 (UCP2), and reducing reactive oxygen species (ROS) production (35, 75). Collectively, these effects support adenosine triphosphate (ATP) production and preserve cardiac homeostasis under hypoxic conditions.

Similarly, Spx levels was remarkably upregulated in other organs post-MI, highlighting its broader role in the systemic response to cardiac ischemia. Fourteen days post-MI, SPX expression was significantly elevated in the brain and liver. In the brain, nerolidol treatment normalized Spx levels while attenuating oxidative stress, inflammation, and metabolic imbalance (76). In the liver, VITD treatment restored Spx levels and alleviated histopathological changes, including liver congestion, sinusoidal dilatation, hepatocyte necrosis, and fibrosis (77). These findings suggest that SPX may function as a local biomarker of ischemic injury and a mediator of tissue-protective responses across multiple organs, potentially linking cardiac events to secondary brain and liver complications.

In contrast, clinical data suggest that circulating SPX may behave differently. In patients with acute MI (AMI), serum SPX levels were significantly lower at admission than those in patients with non-cardiac chest pain, with no significant difference observed between ST-segment elevation myocardial infarction (STEMI) and Non-STEMI patients. Receiver operating curve analysis showed a sensitivity of 58%, specificity of 76%, positive predictive value of 82.9%, and negative predictive value of 47.5% at an optimal cutoff of 532 pg/mL for distinguishing AMI from non-cardiac chest pain (78). These findings support the potential utility of serum SPX as a supplementary diagnostic biomarker for AMI.

These seemingly opposing trends, SPX upregulation in cardiac tissues in animal models but downregulation in humans, highlight a need for systematic investigation into the spatial and temporal dynamics of SPX in both myocardial tissue and circulation following MI.

### 3.2 Atrial fibrillation

Integrating clinical observations with mechanistic insights from animal models, recent studies highlight a critical role for SPX in atrial fibrillation (AF). Plasma SPX levels were inversely associated with age, one of the key risk factors for AF (79, 80). Patients with AF exhibited significantly lower plasma SPX levels than those in sinus rhythm, and *Spx* knockout (KO) mice showed increased AF susceptibility. Mechanistically, loss of *Spx* upregulated the expression of potassium inwardly rectifying channel subfamily J member 2 (*KCNJ2*) and sarcolipin (*SLN*), leading to increased Inward Rectifier Potassium Current (I_K1_) and impaired Ca^2+^ handling. Consistently, cardiomyocyte-specific *Galr2* KO mice showed a higher incidence of AF, along with elevated I_K1_ and intracellular Ca^2+^ overload. Additionally, both *Spx*- and *Galr2*-KO mice exhibited increased phosphorylation of cAMP response element-binding protein 1 (CREB1), a transcription factor that binds to the promoter regions of both *KCNJ2* and *SLN*. In agreement, Spx treatment suppressed CREB1 phosphorylation, reduced I_K1_ and Ca^2+^ overload, and decreased AF inducibility in Ang-II-infused mice (80, 81). These findings suggest that SPX reduces AF susceptibility by downregulating KCNJ2 and SLN expression via the SPX/GALR2/CREB1 signaling pathway, highlighting its potential as a therapeutic target for AF.

### 3.3 Diabetes, metabolic syndrome, and metabolic disorder

DM and MS are well-established risk factors for AF and sudden cardiac death (SCD), and they significantly contribute to the development and progression of other CVDs (82–84). Increasing evidence indicates that SPX participates in the pathogenesis of metabolic disorders, with implications for glycemic control, inflammation, and cardiovascular risk.

In adults with prediabetes undergoing a 6-month lifestyle intervention, circulating SPX levels increased significantly only in participants who achieved the greatest reduction in fasting glucose. This increase was observed exclusively in females, with higher post-intervention SPX levels independently and inversely associated with fasting glucose after adjustment for age and BMI, suggesting a sex-specific role for SPX in metabolic adaptation to lifestyle intervention (85). In post-menarcheal adolescent females, circulating SPX levels were unrelated to BMI, body fat, or indices of glucose metabolism, but were directly associated with lipoprotein(a). Notably, SPX levels correlated positively with testosterone and free androgen index in overweight/obese adolescents, whereas in normal-weight adolescents, SPX was negatively associated with dehydroepiandrosterone sulfate (DHEA-S), a steroid hormone primarily produced by the adrenal glands. These findings suggest potential roles for SPX in modulating the reproductive and adrenal axes (86).

Consistent with human data, preclinical models of MS and T2DM demonstrate a significant reduction in circulating SPX levels, supporting its involvement in metabolic regulation. In rats with high-fructose diet (HFD)-induced MS, decreased Spx levels were associated with increased BMI, elevated blood pressure, hyperglycemia, hyperinsulinemia, IR, hyperuricemia, accumulation of advanced glycation end products, and heightened pro-inflammatory markers(IL6 and TNF-α), along with dyslipidemia. Additionally, reductions in peroxisome proliferator-activated receptor gamma (PPARγ) and adenosine monophosphate-activated protein kinase (AMPK) were also noted. Administration of Spx significantly attenuated these metabolic abnormalities (87). Likewise, in a streptozotocin-induced T2DM rat model, Spx levels were decreased, coinciding with increased BMI, serum glucose and insulin, IR, dyslipidemia, inflammatory cytokines, oxidative stress markers, and cardiovascular risk factors such as elevated atherogenic index and MAP. In addition, a decline in β-cell function, HDL cholesterol, and antioxidant enzyme activity (e.g., superoxide dismutase) was also observed. Spx treatment markedly improved these diabetes-induced cardiometabolic, inflammatory, oxidative, and structural impairments, demonstrating efficacy comparable to standard antidiabetic therapies (88).

Although most studies demonstrate an inverse correlation between serum SPX levels and metabolic indexes, variability does exist. In children born small for gestational age (SGA), SPX levels didn't differ from those in peers born appropriate for gestational age (AGA) after adjusting for BMI. However, prepubertal children with at least one MS component exhibited significantly lower SPX levels compared to those without any MS components, regardless of birth weight status (89). In adults, serum SPX levels were significantly lower in individuals with MS compared to non-MS controls after adjustment for age and BMI. However, a sex-stratified analysis revealed that this association was evident only in women, suggesting sex-specific relationships with metabolism (90). Karaca, et al. showed that fasting serum SPX levels were significantly lower in patients with T1DM and T2DM compared to healthy controls, but correlations with other clinical parameters including glycemic or lipid profiles, BMI, cortisol levels, HbA1c, or thyroid-stimulating hormone (TSH) were either inconsistent or absent (62, 91). In a large cohort study, serum SPX levels exhibited a bell-shaped rather than a linear pattern: they were elevated in FDRs of T2DM patients and in individuals with IGR compared to healthy controls, remained high in newly diagnosed T2DM, and significantly declined in established T2DM (64). Interestingly, pregnant women with gestational DM (GDM) exhibited higher serum SPX than healthy controls, with positive correlations to IR (71). These inconsistencies underscore the need for further investigation into the factors influencing SPX levels and their complex relationship with metabolic health. Collectively, preclinical and human evidence suggests that SPX is a key regulator of metabolic homeostasis, with sex-specific effects, and potential as a therapeutic target for MS, T2DM, and cardiovascular diseases.

### 3.4 Inflammation

Inflammation plays a pivotal role in the initiation, progression, and outcomes of CVD (92). SPX exerts potent anti-inflammatory effects by mitigating vascular damage, primarily through suppression of chronic systemic inflammation and vascular calcification, thereby attenuating CVD progression. In an adenine-induced chronic renal failure (CRF) rat model, Spx treatment significantly attenuated CRF-induced upregulation of proinflammatory cytokines (IL-1β, IL-10, IL-17A, and TGF-β1), matrix metalloproteinases (MMP-1, MMP-3, MMP-9, MMP-13, MMP-14), and neutrophil gelatinase-associated lipocalin (NGAL) levels. These molecular changes were accompanied by substantial histopathological improvements in both aortic and cardiac tissues (93). In contrast, chronic SPX treatment aggravated renal IRI in a mouse model by activating the Wnt/β-catenin signaling pathway and promoting inflammation, apoptosis, and fibrosis. Notably, SPX exerted more severe deleterious nephrotoxic effects in healthy kidney than in IRI-injured kidney (94). These contradicting findings likely stem from difference in species, disease models, and treatment regimens, underscoring the context-dependent nature of SPX's renal and cardiovascular effects. VITD offers cardiovascular protective effect through enhancing glutathione synthesis, reducing reactive oxygen species (ROS), and suppressing proinflammatory cytokines via activation of VITD receptor. Notably, in a model of VITD-mediated liver protection after MI, hepatic Spx expression increased after MI (likely as a compensatory response) but was downregulated by VITD treatment, reflecting normalization of endogenous SPX (77). These findings collectively highlight SPX's dual potential as a therapeutic agent and diagnostic biomarker in CVD and related hepatic complications.

### 3.5 Chemotherapy-induced cardiotoxicity

Doxorubicin (DOX) is a highly effective chemotherapeutic agent used in a wide range of tumors; however, its dose-dependent cardiotoxicity poses a major limitation to its clinical use, underscoring the urgent need for novel therapeutic strategies that preserve cardiac function without diminishing its anticancer efficacy (95, 96). In a DOX-induced cardiotoxicity mouse model, Spx treatment improved cardiac function and attenuated cardiotoxicity by reducing iron accumulation and abnormal lipid metabolism, as well as by inhibiting excessive autophagy-induced ferroptosis (97). Mechanistically, this cardiac protective effect was mediated by Beclin 1, a key autophagy regulator, since knockdown Beclin 1 eliminated these protective effects (97, 98). These preliminary data position SPX as a promising biomarker and therapeutic target for mitigating DOX-induced cardiotoxicity.

### 3.6 Peripheral chemoreception and blood pressure regulation

The carotid body (CB) is a key peripheral chemoreceptor that senses changes in blood oxygen, carbon dioxide, and pH, triggering reflexes that fine-tune ventilation and sympathetic activity to maintain normal blood pressure and oxygen homeostasis. Emerging evidence suggests that SPX may modulate carotid body activity and its response to hypoxia, potentially impacting both respiratory and blood pressure control (99). In the CB, SPX is diffusely expressed in type I cells (glomus cells), but not in type II cells (sustentacular cells) in both humans and rats. This specific distribution supports a modulatory role for SPX in peripheral chemoreception and in the plasticity of the carotid body under hyperoxic conditions. In rats exposed to 60% hyperoxia during the first two weeks after birth followed by four weeks of normoxia, carotid body volume was significantly reduced compared to age-matched normoxic controls, a change that persisted despite the return to normoxia. Notably, *Spx* mRNA expression was 6–7 times higher in hyperoxia-exposed rats, indicating a potential regulatory role in hyperoxia-induced plasticity (25).

Prediabetes and hypertension frequently coexist and represent major risk factors for macrovascular and cardiovascular complications. In hypertensive patients, particularly non-dippers (those whose night-time blood pressure falls by less than 10%), circulating SPX levels were significantly lower compared to healthy individuals. These patients also exhibited increased left atrial volume index, interventricular septum thickness, and posterior wall thickness. Notably, SPX levels were negatively correlated with BMI, night-time systolic and diastolic BP, left atrial volume index, and low-density lipoprotein (LDL) cholesterol (100). A similar inverse correlation was observed in children with high body fat mass and elevated SBP compared to those with normal values (45). In rats, HFD induced MS elevated BP, an effect attenuated by Spx treatment (87). Together, these findings position SPX as a promising theranostic marker for hypertension in individuals with MS and prediabetes.

## 4 Perspectives

**Figure 1:**
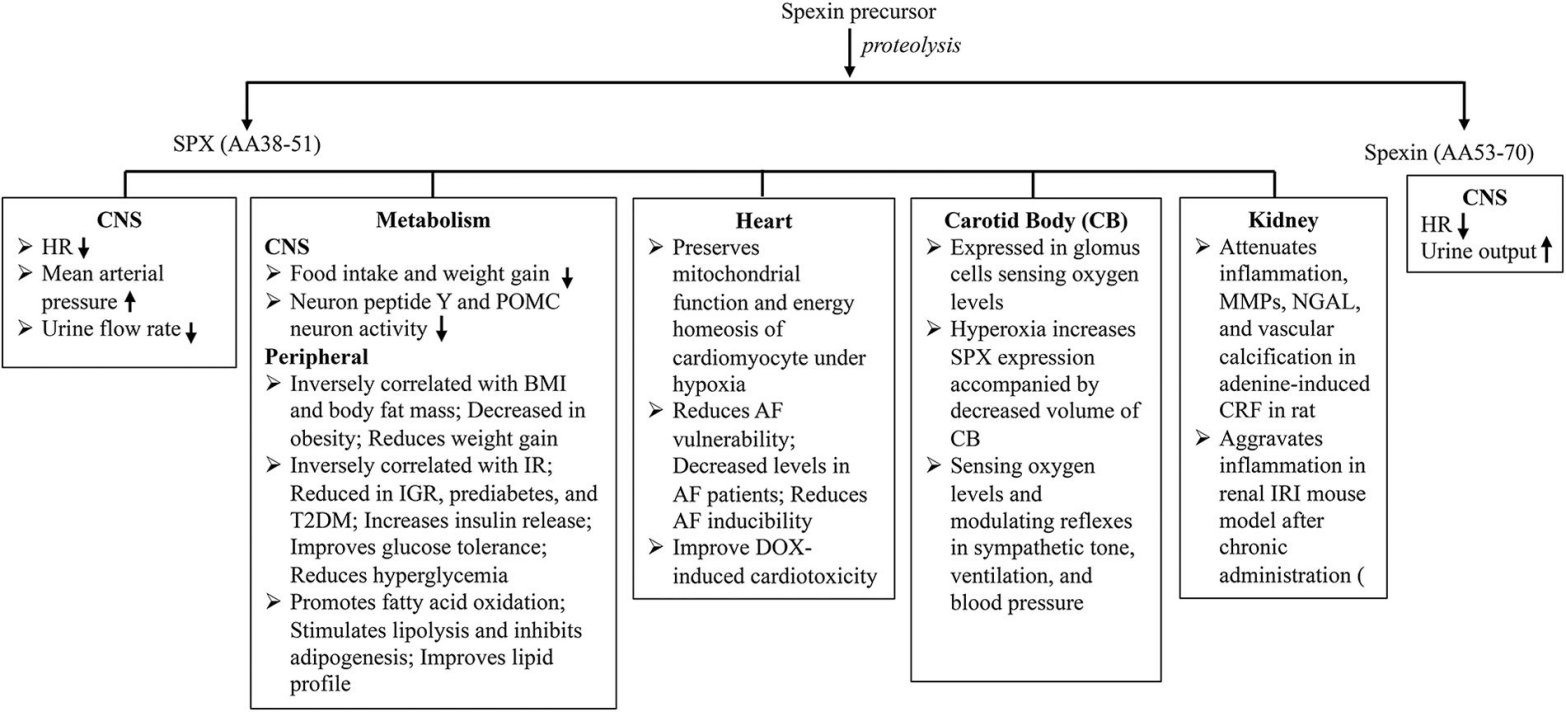
Overview of the multifaceted roles of SPX in cardiometabolic homeostasis. The diagram highlights the primary effects of SPX across the central nervous system (CNS), metabolism, heart, carotid body, and kidney, illustrating its predominantly protective contributions to autonomic regulation, energy balance, cardiac function, chemoreception, and modulation of renal inflammation.

Accumulating evidence indicates that SPX plays a vital role in maintaining cardiovascular homeostasis through both central and peripheral mechanisms. Reported functions include central autonomic regulation of cardiovascular activity and fluid homeostasis, modulation of vascular biology, lipid and glucose metabolism, cellular energy production, carotid body–mediated chemosensory and autonomic responses, and regulation of inflammatory pathways, among others (5, 12, 14, 25–27, 88, 93). These diverse pathophysiological actions collectively contribute to SPX's protective effects on cardiovascular homeostasis. Beyond mature SPX, a distinct proteolytically processed spexin (AA53–70) peptide also contributes to cardiovascular homeostasis (29), as summarized in Figure 1. It is important to note that most of those findings are based on epidemiological, cross-sectional, or descriptive experimental studies, and direct causal relationships between SPX and cardiovascular outcomes have not yet been firmly established. That being said, SPX has been shown to be inversely correlated with obesity, MS, IR, and elevated proinflammatory markers, all well-established cardiovascular risk factors. Consistently, lifestyle interventions have been shown to increase circulating SPX levels, accompanied by improved metabolic profiles. Moreover, reduced circulating SPX levels have been observed in patients with AMI and AF, compared to their respective control groups (78, 80, 81). These clinical observational results are supported by *in vitro* and preclinical experimental studies (35, 74, 80, 97). These discoveries provide a solid foundation for further investigation into the molecular mechanisms underlying the cardiometabolic and cardioprotective actions of SPX and highlight its potential as a therapeutic target for cardiovascular disease prevention and treatment.

Notably, sex differences in cardiovascular disease prevalence, pathogenesis, and response to therapy, have been well recognized, yet the underlying molecular mechanisms remain incompletely understood. Biological differences in sex hormones, body fat distribution, lipid metabolism, and inflammatory signaling contribute to distinct cardiovascular risk profiles in males and females (101–103). Emerging evidence suggests that SPX may be one of the gender-specific factors contributing to these sex differences in cardiometabolic profiles. In adults with MS, circulating SPX levels were lower than in those without MS, with the association primarily observed in women (85, 86, 90). Collectively, these findings suggest that SPX may play a sex-specific role in metabolic and cardiovascular risk and that modulating SPX levels through lifestyle or therapeutic interventions could provide a novel, sex-tailored strategy for cardiovascular disease diagnosis, prevention and management.

To fully realize the theranostic potential of SPX, several critical knowledge gaps must be addressed: (1) identification of the full spectrum of spexin precursor-derived small peptides, their cognate receptors, biological functions, and downstream signaling pathways; (2) clarification of the intracellular signaling cascades downstream of GALR2 and GALR3 in cardiovascular tissues; (3) comprehensive characterization of the full repertoire of SPX receptors other than CALRs, along with their regulatory mechanisms under both physiological and pathological conditions; (4) resolution of inconsistencies observed across studies, including variations between animals (mouse vs. rat), disease models, species (human vs. preclinical), as well as sex-related variability in reported associations between circulating SPX levels and metabolic/cardiovascular diseases; (5) determination of whether alterations in SPX levels are causative, compensatory, or incidental in cardiovascular pathology, with attention to sex-specific effects; and (6) implementation of larger, longitudinal human studies integrating SPX with cardiometabolic profiling and sex-stratified analyses to enhance translational relevance. Addressing these gaps through coordinated basic, translational, and clinical research efforts will be essential to advancing SPX as a viable target for the prevention and treatment of CVD.
